# Porous polylactic acid scaffolds for bone regeneration: A study of additively manufactured triply periodic minimal surfaces and their osteogenic potential

**DOI:** 10.1177/2041731420956541

**Published:** 2020-11-06

**Authors:** Anna Diez-Escudero, Hugo Harlin, Per Isaksson, Cecilia Persson

**Affiliations:** 1 Division of Applied Materials Science, Department of Materials Science and Engineering, Uppsala University, Uppsala, Sweden; 2 Division of Applied Mechanics, Department of Materials Science and Engineering, Uppsala University, Uppsala, Sweden

**Keywords:** Additive manufacturing, triply periodic minimal surfaces, polylactic acid, bone scaffolds

## Abstract

Three different triply periodic minimal surfaces (TPMS) with three levels of porosity within those of cancellous bone were investigated as potential bone scaffolds. TPMS have emerged as potential designs to resemble the complex mechanical and mass transport properties of bone. Diamond, Schwarz, and Gyroid structures were 3D printed in polylactic acid, a resorbable medical grade material. The 3D printed structures were investigated for printing feasibility, and assessed by morphometric studies. Mechanical properties and permeability investigations resulted in similar values to cancellous bone. The morphometric analyses showed three different patterns of pore distribution: mono-, bi-, and multimodal pores. Subsequently, biological activity investigated with pre-osteoblastic cell lines showed no signs of cytotoxicity, and the scaffolds supported cell proliferation up to 3 weeks. Cell differentiation investigated by alkaline phosphatase showed an improvement for higher porosities and multimodal pore distributions, suggesting a higher dependency on pore distribution and size than the level of interconnectivity.

## Introduction

Biomaterials and structures that characteristically mimic bone in its entirety are still a challenge. The complexity of bone tissue not only lies in its compositional properties but also in its highly hierarchical structure combined with several levels of porosity. Successful bone grafts are often considered to be biocompatible, bioactive, porous and permeable to allow nutrient supply and cell colonization, and mechanically stable.^1,2^ Fulfilling all these requirements in one scaffold remains elusive; however, additive manufacturing is a promising technique to overcome some of the design challenges due to the ability to closely control relevant parameters such as porosity and structural integrity.

Bone grafts for tissue engineering are intended to guide and support the bone’s self-healing capacity when it is compromised, and in some cases, to stimulate and foster the bone healing process. In order to provide cell instructive materials, various routes have been investigated that focus on tuning physical and chemical cues of the materials, such as surface chemistry or topography,^3^ substrate stiffness,^4,5^ surface curvature,^6^ porosity and pore sizes, and geometry.^7^ Although the crucial role of porosity, pore size, and architecture on bone graft performance is widely accepted,^8,9^ these parameters also affect a cascade of biological events such as cell and nutrient diffusion and migration.^10^ Despite the vast literature on parameters affecting bone graft performance both in vitro and in vivo, it is difficult to establish a straightforward convention for optimal design due to the lack of comparable studies and the multiparametric nature of bone scaffold design.

A strategy to overcome the complexity of bone morphology is to use mathematical models to design complex architectures that mimic some of the bone features such as porosity, pore architecture, curvature, and interconnectivity. The development of high-resolution imaging techniques has also enabled the characterization of such parameters. For instance, Jinnai et al. exhaustively characterized the curvature of bone microarchitecture pointing to an overall mean curvature close to zero.^11^ Shi et al. applied mathematical models to reproduce cancellous bone structures from rabbit condyles using so-called minimal surface techniques which closely resemble bone architecture.^12^ The technique has attracted great interest as nature-inspired models applied to bone scaffolding due to their limited surface curvature similar to trabeculae. Further development of additive manufacturing (AM) techniques has enabled the implementation of such complex designs into real scaffolding. AM technology allows a close control of scaffold geometry with high reproducibility and relatively low production costs.

The use of triply periodic minimal surfaces (TPMS) as bone constructs using mainly metals and polymers has been reported. Yan et al. developed porous titanium alloy implants through selective laser melting (SLM) achieving compatible mechanical properties with trabecular bone providing minimized stress-shielding effects for prosthetics.^13^ Likewise, Bobbert et al. applied TPMS designs on titanium alloys that mimic the topological, mechanical, and mass transport properties of bone.^14^ Some researchers have also applied TPMS designs using polymers, mainly based on acrylates, polyamides or styrenes through stereolitography (SLA), to investigate the mass transport or mechanical properties of the structures.^15–18^

While the above polymer- or metallic-based TPMS structures might show biocompatibility, few studies have exploited the use of resorbable polymers which can serve as temporary constructs and further integrate into the bone remodelling process. In the present work, polylactic acid (PLA) was investigated as substrate material to manufacture TPMS scaffolds through fused deposition modelling (FDM). PLA is an aliphatic polyester widely used for biomedical devices due to its good biocompatibility, processability, and degradation rates.^19^ The thermoplastic behavior of PLA has enabled its use in AM through the FDM technique, which stands as the lowest cost and quickest technology. Hence, in this study, FDM technique was used to manufacture TPMS scaffolds using commercial PLA filament. The mass transport and mechanical properties, morphology and porosity, and the biological behavior of the TPMS scaffolds were evaluated as potential bone regenerative scaffolds.

## Materials and methods

### Scaffold design

Three different TPMS scaffolds were designed using the commercial code Matlab (The MathWorks, Inc., Natick, MA, USA). Isosurfaces of the TPMS were generated using equations (1)–(3)^20,21^:

(1) $$\begin{array}{l} \Gamma_{D}=\sin[\lambda_{x}x]\sin[\lambda_{y}y]\sin[\lambda_{z}z]\\ \;\;\;\;\;\;\;\;\;\;\;\;\;\;\;\;\;\;\;\;\;\;+\sin[\lambda_{x}x]\cos[\lambda_{y}y]\cos[\lambda_{z}z]+\\ \;\;\;\;\;\;\;\;\;\;\;\;\;+\cos[\lambda_{x}x]\sin[\lambda_{y}y]\cos[\lambda_{z}z]\\ \;\;\;\;\;\;\;\;\;\;\;+\cos[\lambda_{x}x]\cos[\lambda_{y}y]\sin[\lambda_{z}z]-C_{D}, \end{array}$$

(2) $$\Gamma_{S}=\cos[\lambda_{x}x]+\cos[\lambda_{y}y]+\cos[\lambda_{z}z]-C_{S},\;\;\;and$$

(3) $$\begin{array}{l} \Gamma_{G}=\cos[\lambda_{x}x]\sin[\lambda_{y}y]+\cos[\lambda_{y}y]\sin[\lambda_{z}z]\\ \;\;\;\;\;\;\;\;\;\;\;+\sin[\lambda_{x}x]\cos[\lambda_{z}z]-C_{G}. \end{array}$$

Above, subscripts D, S, and G refer to geometries Diamond, Schwarz, and Gyroid, respectively, while Γ denotes the isosurface, and the parameter C control the structure’s porosity. The periodicities of the structures are determined by the wavenumber $\lambda_{i}=2\pi n_{i}/L_{i}$, where i=x,y,z and $n_{i}$ is the number of cell repetitions along each spatial direction of length $L_{i}$.

**Figure 1.**
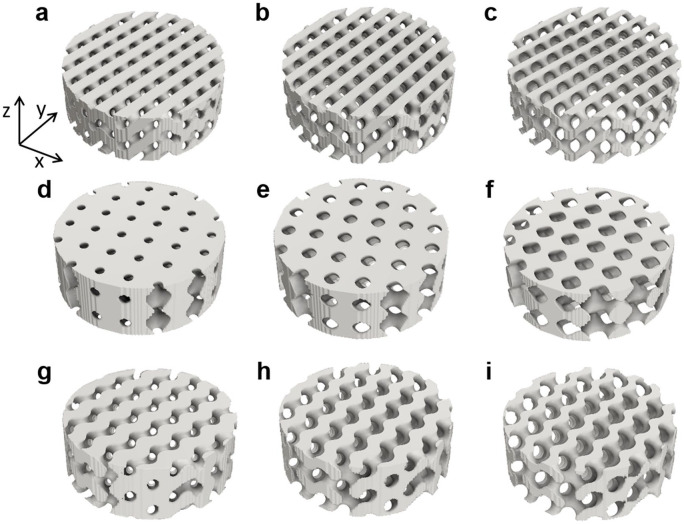
TPMS structures with different levels of porosity, obtained by equations (1)–(3). Diamond with increasing porosity 35%, 50%, and 65% and C_D_ values of −0.3643, 0, and 0.364, respectively (a, b, c). Schwarz structures of 35%, 50%, and 65% porosity obtained with C_S_ values of −0.463, 0, and 0.463, respectively (d, e, f). Gyroid structures of 35%, 50%, and 65% porosity with C_G_ values of −0.5242, 0, and 0.5244, respectively (g, h, i). A Cartesian coordinate system (x, y, z) is attached to the geometries.

Structures of 4.8 × 4.8 × 9.6 and 6 × 6 × 2 unit cells, with sizes of 12 mm diameter and 24 mm height, and 15 mm diameter and 5 mm height were used. Figure 1 shows some geometries with different levels of porosity ranging within those of trabecular bone.

### Printing technique

A FDM technique was used to manufacture the triply periodic minimal surface-based scaffolds. In this procedure, a thermoplastic filament is extruded through a heated nozzle that melts the polymer filament and deposits it on a buildplate which is often heated to temperatures close to the glass transition temperature. An Ultimaker S5 printer (3DVerkstan AB, Sweden) and a commercial 2.85 mm thickness PLA transparent filament (3DVerkstan AB, Sweden) were used to obtain the final TPMS structures using a 0.25 mm nozzle. The specific parameters defined for printing are summarized in Table 1.

**Table 1.** Printing parameters.

FDM parameters	
Layer height	0.06 mm
Line width	0.23 mm
Infill	Lines 100%
Printing temperature	190 °C
Buildplate temperature	60 °C
Print speed	30 mm/s

### Chemical and thermal characterization

Differential scanning calorimetry (DSC) and thermogravimetric (TGA) analyses were performed using a TA instrument DSC Q2000 V24 and TGA Q500 V20 (TA Instruments, Newcastle, DE, USA).

Samples for DSC were analyzed through a heat/cool/heat method: from room temperature to 220°C a heating rate of 10°C/min was used, a cooling down to 0°C at 5°C/min was then applied, and finally, heating up to 220°C at 10°C/min. Samples of 7.5 ± 0.1 mg consisting of PLA before and after printing were sealed into aluminium pans. All experiments were conducted in nitrogen at a flow rate of 50 mL/min. The degree of crystallinity before and after the printing process was calculated using equation (4).^22^

(4) $$X_{c}=\frac{\Delta H_{m}-\Delta H_{C}}{\Delta H_{m}^{0}},$$

where ∆Hm is the melting enthalpy, calculated by integrating the peak area of the melting transition (onset temperature 144°C, offset temperature 164°C, for before printing, and 146°C and 163°C, for after printing respectively), ∆HC is the crystallization enthalpy (onset temperature 106°C, offset temperature 142°C, for before printing, and 117°C and 143°C, for after printing, respectively), and ∆H0m is the enthalpy of 100% crystalline PLA, reported in literature as 93.7 J/g.^23^

TGA analyses were conducted from room temperature up to 600°C at a heating rate of 10°C/min under nitrogen flow (50 mL/min), using approximately 15 mg of the samples. The degradation temperature was estimated from the weight loss-temperature plots.

Attenuated total reflectance-Fourier infrared spectroscopy (ATR-FTIR) was carried out on as-received filaments and processed 3D-printed scaffolds to investigate any chemical transformation during the printing process. ATR-FTIR was measured in a Tensor 27 spectrometer equipped with KBr beamsplitter and RT-DLaTGS detector (Bruker Ettlingen, Germany). Spectra were acquired using 256 scans with a resolution of 4 cm^−1^ in the range of 4000 to 400 cm^−1^ with a diamond crystal.

### Morphological and structural characterization

Micro-computed tomography (SkyScan 1172, Bruker, Kontich, Belgium) was used to quantify the porosity. Images were acquired using a voxel size of 20 µm, a Cu-Al filter at a voltage of 60 kV and a current of 162 µA. Four specimens of each geometry and porosity were analyzed. Reconstruction of cross sections was done using software package NRecon (Bruker micro-CT, Kontich, Belgium). Calculations of macroporosity and pore size distributions were performed with CTAn (Bruker, Kontich, Belgium). The interconnectivity of the scaffolds was quantified by the Euler number, which expresses the number of connections in a given structure^24^ obtained from micro-CT calculations. The surface morphology of the printed TPMS-PLA scaffolds was investigated by scanning electron microscopy (SEM, Merlin Zeiss, Germany).

Compression tests were used to investigate the mechanical properties. Cylinders of 12 mm diameter and 24 mm height were tested in the axial direction using a tensile testing machine (Shimadzu AGS-X, Shimadzu Europe GmBH, Germany) at a constant displacement speed of 1 mm/min, using 5 and 10 kN load cells. Both geometry and porosity were tested using six specimens each. Prior to testing, the scaffolds were embedded in rapid curing resin to provide parallel end surfaces. The structures’ global Young’s modulus was calculated from the slope within the linear regime of the stress-strain curves, and the compressive strength was estimated from the global peak.

**Figure 2.**
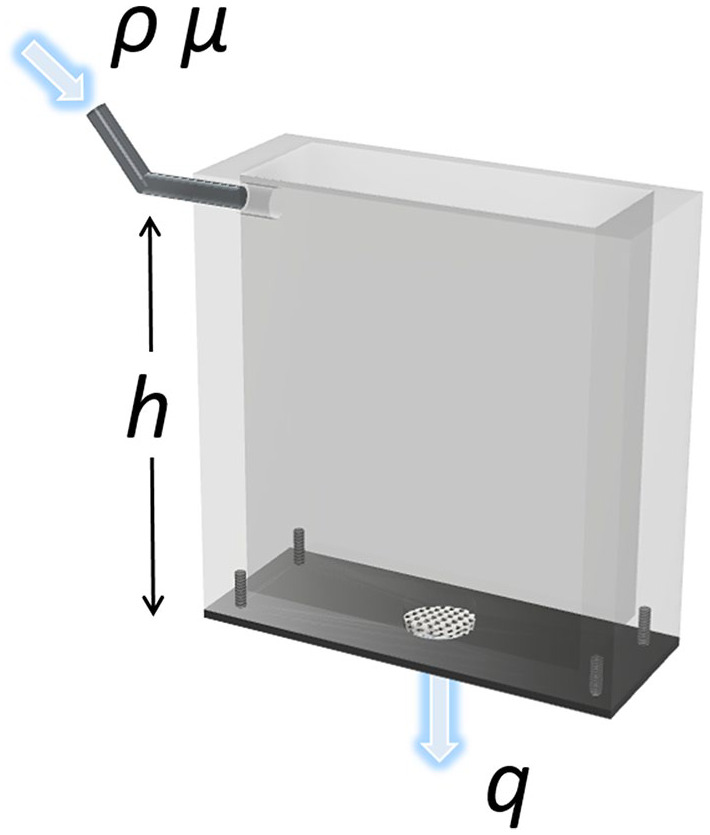
Illustration of the permeability box showing the sample placement, inlet for the BMF fluid and outlet for readout of fluid flow rate.

Longitudinal permeability was investigated using a blood mimicking fluid (BMF) adapted from literature.^25^ Briefly, an aqueous solution with glycerol, dextran, and poloxamer was prepared, resulting in a fluid with a density of 1037 kg/m^3^ and a dynamic viscosity of 4 mPa·s. A custom-made setup to measure the longitudinal permeability of the scaffolds (15 × 5 mm^2^) was 3D printed for that purpose (Figure 2).

**Figure 3.**
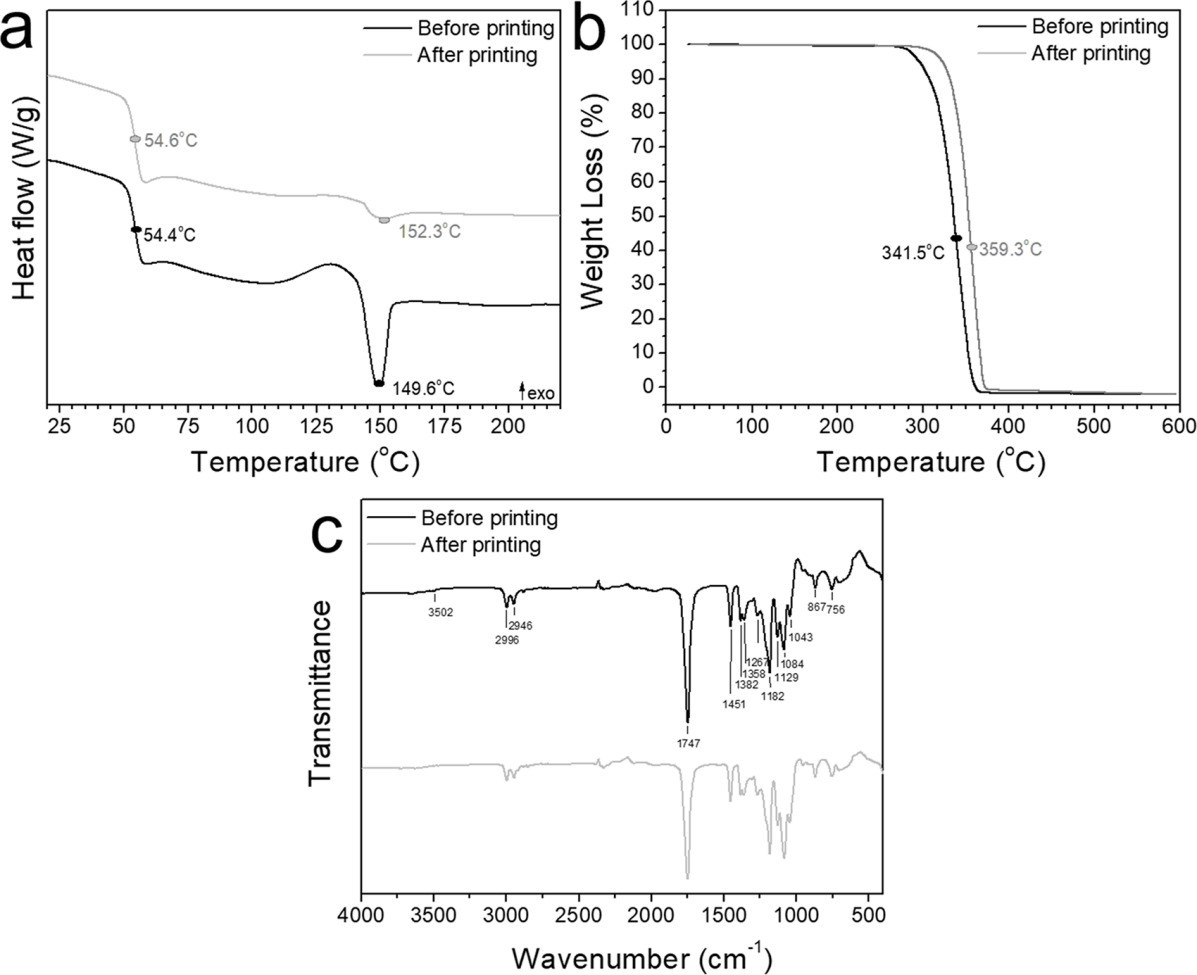
DSC (a), TGA (b), and ATR-FTIR (c) analyses of PLA before (black) and after printing (gray) depicting the thermal characteristics; a: glass transition temperature, recrystallization and melting temperature, b: degradation temperature, c: typical bands corresponding to PLA composition.

The scaffolds were laterally sealed to ensure only longitudinal flow of the BMF. The tank was filled with BMF at a constant height, and the fluid passing through the porous scaffold was collected and time recorded; all tests were performed at room temperature, and four specimens of each geometry and porosity were tested. The BMF height corresponded to 30 mm, which yielded a pressure difference of 305 Pa according to:

(5) Δp=ρgh,

where *Δp* is the pressure difference, *ρ* is the density of the BMF (1.037 g/cm^3^) and *h* is the height of the fluid (Figure 3). To calculate the permeability of the porous media, a simplified version of Darcy’s law was used:^26^

(6) $$K=\mu qL_{z}/\left[A\Delta p\right],$$

where K is the longitudinal permeability, µ is the viscosity of the BMF, q is the fluid flow rate, A is the cross-sectional area of the scaffold and $L_{z}$ is the length of the scaffold in the axial direction (Figure 1).

### Biological characterization

Osteoblast-like cells (SaOs-2, ECACC Sigma Aldrich, United Kingdom, passage 19) were used to investigate the cell viability, proliferation, and differentiation on TPMS-PLA scaffolds. All scaffolds were sterilized using 70% ethanol immersion for 3 h, followed by three rinses in phosphate-buffered saline (PBS, Sigma Aldrich, St. Louis, USA). Afterwards, complete cell medium was supplemented and left overnight for pre-conditioning. Cell medium consisting of Dulbecco’s Modified Eagle Medium (DMEM) was supplemented with 10% fetal bovine serum (FBS), 2% 4-(2-hydroxyethyl)-1-piperazineethanesulfonic acid (HEPES), 1% sodium pyruvate and 1% penicillin/streptomycin (all from Sigma Aldrich) and considered as complete DMEM. After pre-conditioning, cells were seeded at a density of 5·10^4^ cells/mL. In order to optimize cell adhesion onto the scaffolds, cells were seeded as a concentrated cell drop of 20 µL, and complete medium was added (20 µL) every 15 min until reaching the complete volume (1 mL).

Protein adsorption was studied on the cell culture media supernatants. Supernatants at 1 and 2 days were collected and analyzed for total protein content according to manufacturer’s specifications (Pierce™ Coomassie Bradford Protein Assay Kit, Thermofisher, Germany).

Cell studies were performed over 21 days, and proliferation was assessed at 1, 3, 7, 10, 14, and 21 days. Glass cover slips were used as controls. Alkaline phosphatase (ALP, Sigma Aldrich, St. Louis, USA) was studied in cell lysates to identify differentiation at 1, 7, 14, and 21 days. The total ALP expression was normalized by the total protein in cell lysates measured by Coomasie Protein Assay Kit. A calibration curve consisting of different concentrations from 0.02 to 1 mM of p-nitrophenol phosphate (p-NPP, Sigma-Aldrich, St. Louis, USA) was used to extrapolate the values of ALP to concentration units.

Fluorescent staining was used to visualize the cell nuclei and cytosol using 4′,6-diamidino-2-phenylindole (DAPI, Invitrogen, Massachusetts, USA) and carboxyfluorescein diacetate (CFDA, Sigma-Aldrich, St. Louis, USA), respectively, at 7, 14, and 21 days of cell culture. The samples were rinsed with PBS (×3) and fixed using 2.5% (v/v) glutaraldehyde for 2 h at 4°C. The fixed samples were rinsed in PBS (×3) for 5 min and permeabilized using 0.1% Triton X-100 (Sigma-Aldrich, St. Louis, USA) in PBS for 20 min at room temperature. Subsequently, CFDA solution (500 nM, 400 µL, Sigma-Aldrich) was added and incubated for 10 min at room temperature, followed by three rinses with PBS. Afterward, DAPI solution (300 nM, 400 µL, Invitrogen) was added and incubated in the dark for 5 min, followed by PBS rinsing (×3) prior to imaging using fluorescence microscope (Nikon Instruments Europe B.V., Netherlands). Images were acquired and processed using NIS V4.50 software.

One representative sample for each geometry and porosity was used for SEM after 14 days of cell culture. Cells were fixed using 2.5% of glutaraldehyde in PBS for 2 h at 4°C and consequently rinsed with PBS thrice for 15 min each. Finally, fixed cells were dehydrated gradually in ethanol-water mixtures and a final hexamethyldisilazane (HMDS, Sigma Aldrich, St. Louis, USA) step was added as drying agent. Prior to SEM observation, samples were sputtered a ~5 nm thickness coating of Au-Pd to improve conductivity and reduce thermally-induced specimen damage.

### Statistics

All statistical analyses were performed using IBM SPSS statistics software (IBM Corp. Released 2015. IBM SPSS Statistics for Windows, Version 23.0. Armonk, NY: IBM Corp., USA). One-way ANOVA was used to assess statistical differences between groups. Statistical significance was noted at *p* < 0.05. Homogeneity of variance was assessed by Levene’s test, and Scheffe’s post-hoc test was applied in case of significance (which was always the case).

## Results

### Chemical and thermal characterization

Thermal analyses of PLA both before and after the printing showed a very similar glass transition temperature of 54.4°C versus 54.6°C (Figure 3(a)), regardless of the printing process. Larger differences were found for the recrystallization and melting temperatures, together with the corresponding enthalpies (Table 2). A loss in crystallinity is shown from the smaller peak area for the melting temperature correlated to the degree of crystallinity, equation (4).

**Table 2.** Thermal properties analyzed by DSC of PLA before and after printing (BP and AP, respectively).

Sample	*T_g_* [°C]	*T_m_* [°C]	*T_cc_* [°C]	*ΔH_m_* [J/g]	*ΔH_c_* [J/g]	*Χ_x_* [J/g]
PLA BP	54.4	149.6	130.4	32.0	−70.4	36.3
PLA AP	54.6	152.3	129.4	16.4	−33.4	16.0

Despite the changes in the degree of crystallinity, no further degradation by-products arose due to the printing process as illustrated by the chemical composition analysed by FTIR-ATR, showing identical spectra for both PLA before and after printing (Figure 3(c)). Typical bands at 2996, 2946 cm^−1^ were attributed to the -CH- stretching vibration.^27^ A small shoulder appears at 3502 cm^−1^ corresponding to O-H stretching. Carbonyl stretching appears at 1747 cm^−1^ and C-O corresponding to CH-O is identified at 1182 cm^−1^. The other C-O vibration corresponding to the O-C=O group are assigned at 1129, 1084, and 1044 cm^−1^.^28^ All bands appear in both spectra regardless of the processing thus ensuring no chemical decomposition occurred due to the melting process during printing.

### Morphological and structural characterization

**Figure 4.**
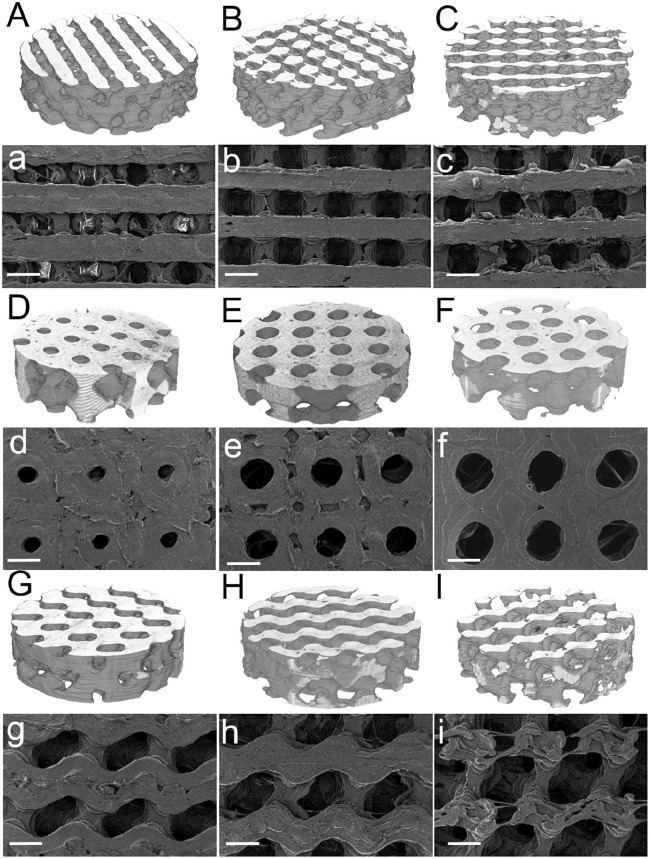
Micro-CT (upper case letters) and SEM images (lower case letters; scale bar 1 mm) of the TPMS Diamond (A, B, and C for micro-CT reconstructions; a, b, and c for SEM surface images; 35%, 50%, and 65% porosity, respectively), Schwarz (D, E, and F, micro-CT 35%, 50%, and 65%, respectively; d, e, and f for 35%, 50%, and 65% porosity SEM surface images, respectively); and Gyroid (G, H, and I; g, h, and i, for micro-CT and SEM respectively, for 35%, 50%, and 65%, respectively).

The micro-CT reconstructions (Figure 4, upper case letters) evidenced the variation in pore sizes among porosities for the same geometry in accordance with the surface SEM images (lower case letters). Diamond geometries showed square-shaped pores (Figure 4(a)–(c)), Schwarz geometries rendered perfectly spherical pores, and Gyroid exhibited ellipsoidal pores. As porosity increased, regardless of the type of geometry, the pore sizes increased.

**Figure 5.**
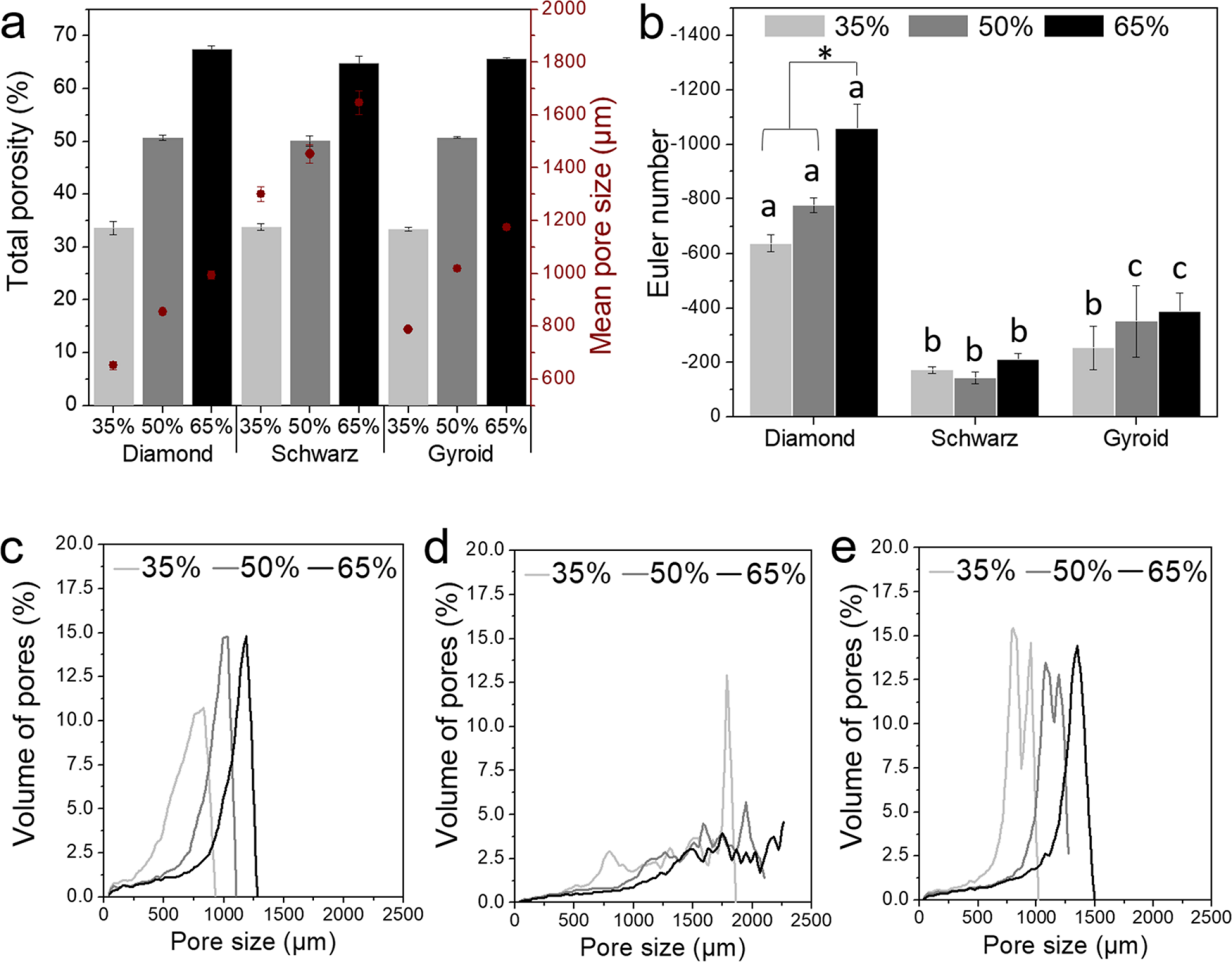
Total porosity (bars) and mean pore sizes (red dots) analysed by micro-CT (a), interconnectivity measured through Euler number (b), statistical significance (*p* < 0.05) denoted by letters for geometries and * for porosities, and pore size distributions for Diamond (c), Schwarz (d), and Gyroid (e). All values were statistically significant (*p* < 0.05).

The porosity quantified by micro-CT showed highly accurate experimental values for the theoretical values implemented in the isosurface equations (equations (1)–(3) and Figure 5(a)). Schwarz geometries depicted the highest mean pore sizes with an overall increase of 100 µm for each level of porosity. Gyroid structures exhibited mean pore sizes ranging approximately from 800 to 1200 µm, from 788 µm for 35%, 1020 µm for 50%, and 1175 µm for 65% porosity. Diamond structures yielded the lowest mean pore sizes ranging from 650 to 1000 µm. The Euler number obtained from the micro-CT scans was used as an indicator of the level of interconnection of the 3D structure.^24,29,30^ The increase in interconnectivity correlated to the increase in the porosity (Figure 5(b)). Diamond structures exhibited the highest interconnectivity, followed by Gyroid, then Schwarz. Three different pore size distributions were produced depending on the geometry used; Diamond exhibited monomodal pore distributions for all porosities (Figure 5(c)), Schwarz, multimodal pore distributions (Figure 5(d)), while Gyroid showed bimodal pore distributions (Figure 5(e)).

**Figure 6.**
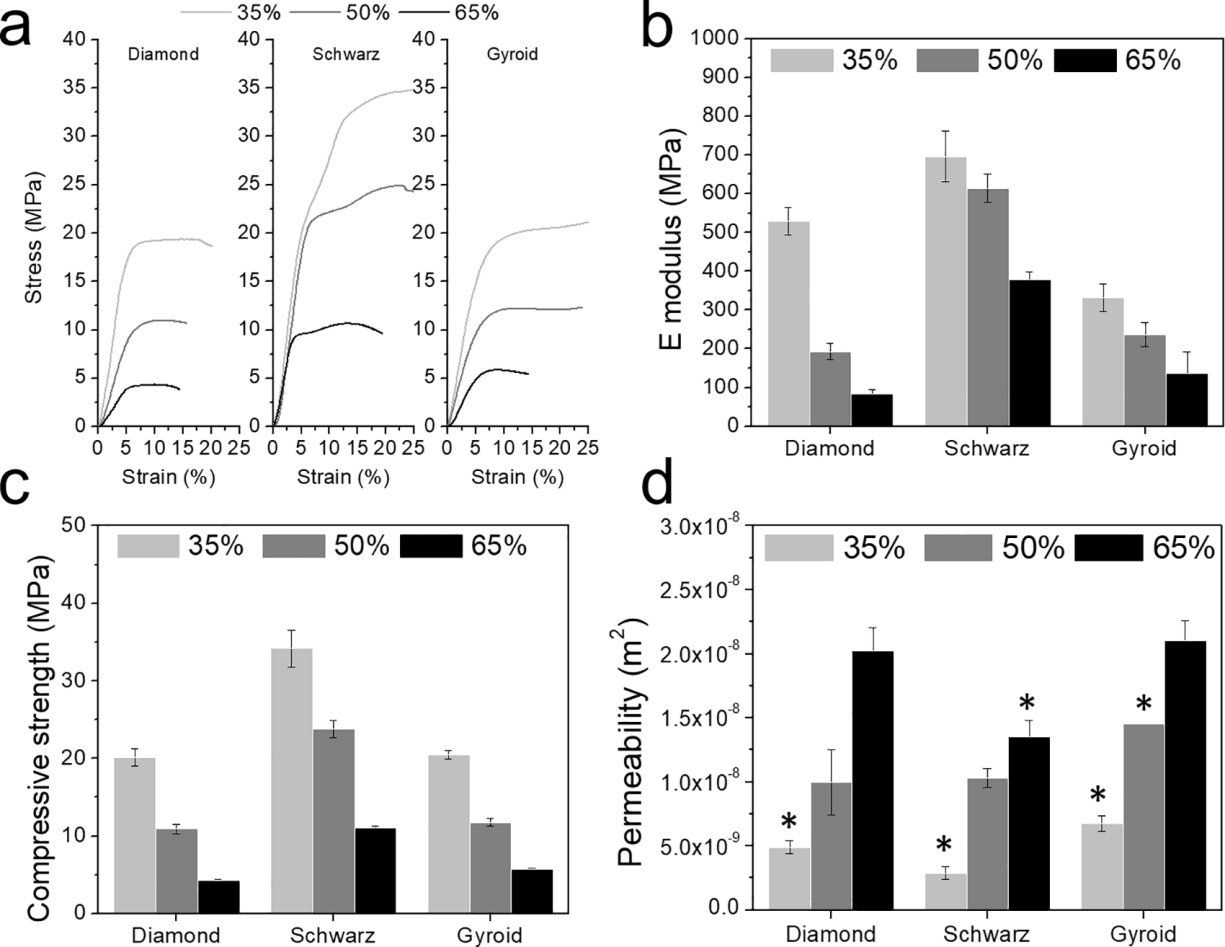
Representative global stress-strain curves obtained in compression experiments (a), Global elastic moduli (b), compressive strength (c), and the permeability values (d) for all porosities and geometries. For b and c all values showed statistical significance (*p* < 0.05). For d, * denotes statistical significance comparing different geometries for the same porosity (*p* < 0.05).

Global stress-strain curves showed a decay in structural stiffness as porosity increases (Figure 6(a)). An overall decrease in the elastic moduli for all geometries as porosity increased was observed, with a larger absolute decrease for Diamond geometry (Figure 6(b)). Similarly, the compressive strength decreased with the increase in porosity, also depicting higher strength for Schwarz geometry (Figure 6(c)). Oppositely, and as expected, permeability values (Figure 6(d)) showed an increase as porosity increased. The highest permeability values were found for Diamond and Gyroid structures.

### Biological characterization

**Figure 7.**
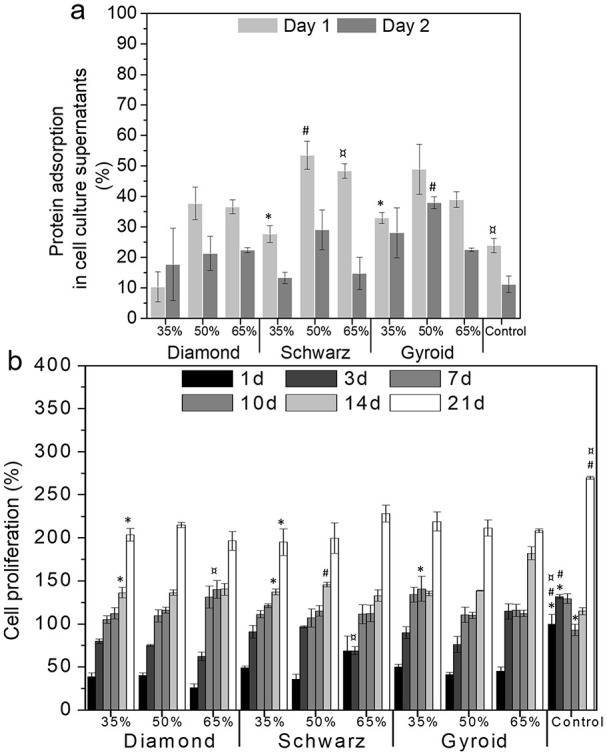
Protein adsorption (a) at early time points (1 and 2 days), cell proliferation over 21 days (b) normalized by TCPS at 1 day of cell culture. Statistical significance between geometries at each time point and same porosity (*p* < 0.05) is denoted with * for 35%, # for 50%, and ¤ for 65%.

Protein adsorption measured indirectly in supernatants at day 1, corresponding to preconditioning, and at day 2, after cell seeding, exhibited an increase in adsorption linearly correlated to the total porosity (Figure 7(a)). Schwarz and Gyroid geometries showed higher adsorption for 50% porosities for both 1 and 2 days.

Lower cell viability was found at early stages (1 day) for all scaffolds as a result of a lower seeding efficiency (Figure 7(b)). Denser structures yielded higher cell adhesion at 1 day compared to 50% and 65% porosity; however, all geometries allowed cell proliferation after 3 days of culture, showing a viability above 75%, except for 65% porosity. Higher porosity structures yielded cell viabilities above 100% after 7 days of cell culture. Over time, all structures showed an increase in cell proliferation which was more pronounced for 50% and 65% porosities.

**Figure 8.**
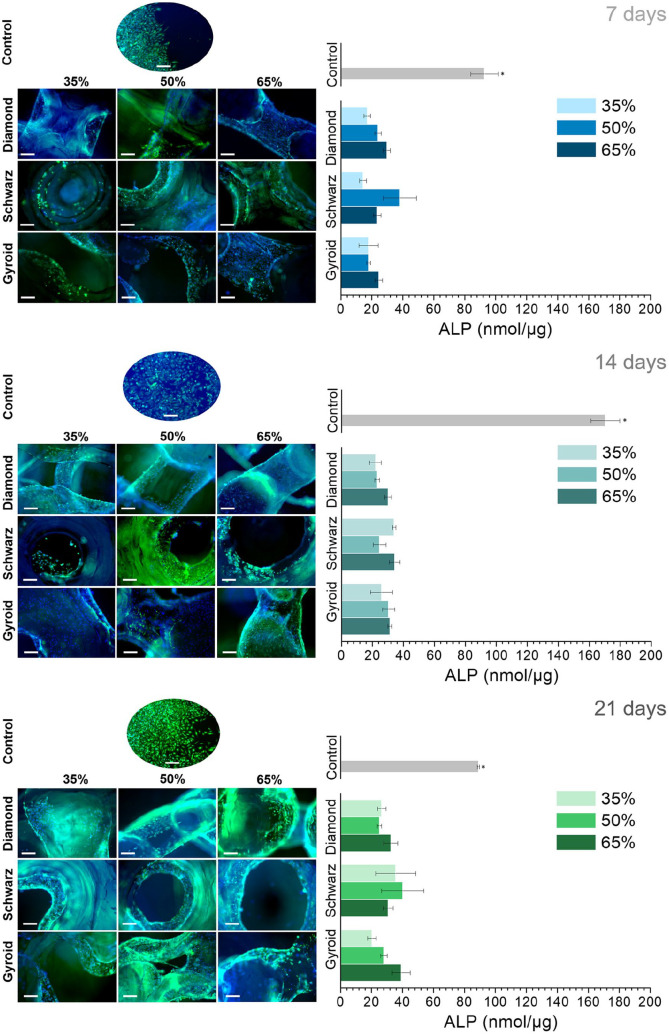
Fluorescent stained images of osteoblast-like cells cultured on the different geometries and porosities over 7, 14, and 21 days (left, scale bar: 100 µm) and the ALP expression at the same time points (right). * denotes statistical significance (*p* < 0.05) between geometries at each time point for the same porosity.

The immunostaining images for each geometry and porosities (Figure 8) depicted a high number of cells distributed along the edges of the printed struts. The ALP analyses (Figure 8, right) along with the fluorescence images demonstrated an increase in ALP expression over time. At 7 days, all geometries showed higher ALP for 65% porosities compared to 35%. This trend was maintained at 14 and 21 days, except for Schwarz 50% and 65%, respectively. Cells on Schwarz geometries were mostly concentrated on the pore concavities, while for both Diamond and Gyroid structures cells adhered to the edges of the printed filaments and at both the concavities and convexities of the pore edges. The highest ALP levels were found for Schwarz 50% and 65% geometries, followed by Gyroid 50% and 65%. Despite exhibiting lower ALP values compared to the other geometries, Diamond geometry exhibited a steady and consistent ALP expression over time.

**Figure 9.**
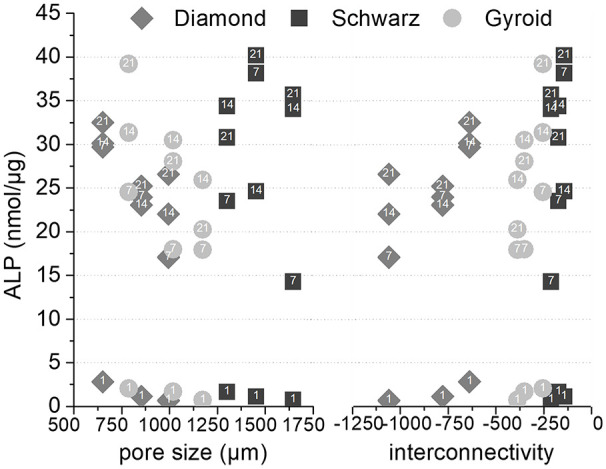
ALP levels as a function of interconnectivity (lower interconnectivity corresponds to higher negative numbers, and higher connectivity corresponds to values closer to zero), and pore size for the three different geometries (number labels in data points refer to the corresponding day of culture).

Further analyses on cell differentiation were performed to correlate the ALP levels with the porosity features analysed through micro-CT analyses, that is, interconnectivity and pore sizes (Figure 9). The lowest values of ALP (<5 nmol/µg) corresponded to the basal levels at day 1 (Supplemental Material, Figure S1). ALP values were higher for larger pore sizes and higher interconnectivity values (Figure 9), and in general, higher for Schwarz geometries, followed by Gyroid. Pore sizes above 1000 µm yielded higher ALP values, while interconnectivity values ranging from −200 to −600 correlated to the highest ALP expression.

## Discussion

Three different TPMS geometries with three levels of porosity, in the range of those of cancellous bone, were investigated as potential bone regenerative scaffolds. A vast number of studies have focused on modelling and studying the mechanical and fluid transport properties of biomorphic TPMS structures through finite element analysis.^18,31,32^ More recently, researchers have explored the use of additive manufacturing techniques to obtain TPMS scaffolds using metals or acrylate-based resins and polyamides.^13,17,33–38^ Despite the efforts toward the implementation of these designs, the authors have found only two studies which have explored the use of resorbable materials as potential tissue engineering scaffolds.^37,39^ Li et al. demonstrated that through topological design using functionally graded scaffolds (FGS), the biodegradation of iron based on Diamond lattices could be adjusted without compromising the biocompatibility of the scaffolds. Interestingly, the mechanical properties such as global elastic modulus and yield strength of the diamond structures retained values within those of trabecular bone after 28 days under dynamic degradation conditions.^37^ Similar Gyroid structures based on PLA obtained through FDM, with 70% to 75% porosity, showed greater mechanical stability than control samples, based on simple struts, highlighting the potential mechanical integrity of TPMS even after degradation.^39^ Given the intrinsic remodelling of bone tissue occurring lifelong, the use of resorbable materials that can be coupled into the bone regenerative process is highly sought after. In this work, a biocompatible and biodegradable polymer, PLA, was used to integrate TPMS designs into FDM printed structures. The scaffolds were characterized for relevant properties in bone tissue engineering, such as porosity, pore size, interconnectivity, mechanical properties, permeability, and cell proliferation and differentiation.

Although PLA has been widely explored as a tissue engineering material,^40,41^ whether its properties remain intact after the printing process is crucial to further understand its potential as a regenerative construct. The chemical and thermal characteristics (Figure 3) demonstrated no structural changes in the PLA backbone structure (Figure 3(c)). Slight differences were obtained in the degree of crystallinity when comparing the material before and after printing. A decrease of 50% in crystallinity was observed after printing correlated to the lower melting enthalpy (Figure 3(a)). Particularly, crystallinity has been shown to play a major role in PLA degradation depicting an inverse relation of the degradation rate with the degree of crystallinity.^42,43^ This decrease in crystallinity combined with the high porosity of the TPMS scaffolds can enhance the degradation rates of pristine PLA, which can vary from 3 months to 2 years.^44,45^

Another key aspect was to confirm that FDM was suitable for the complex geometry of TPMS structures. Several authors have explored various additive manufacturing techniques which provide higher resolution, such as SLM or SLA, compared to FDM technology. The micro-CT reconstructions and SEM images (Figure (4)), together with calculations showed highly reproducible structures in terms of porosity with scarce variation from the theoretical values (equations (1)–(3) and Figure 5(a)). Deeper studies on the morphological properties of the TPMS structures substantiated the differences in pore mean sizes, interconnectivity (Euler number), and pore size distributions. One of the key parameters in bone regenerative medicine is scaffold porosity.^46,47^ Bone possesses a bimodal porosity derived from the combination of cortical or cancellous bone porosity; graded porosity designs have also been explored for the last decade, also with the use of TPMS based designs^33,48–51^ implementing FGS. Worthy to mention is that, despite FGS not being explored in this work, TPMS designs can be easily tuned to allow graded porosities similarly to bone by simply modifying the isosurface equations.^12^

A key parameter related to porosity is the permeability as it allows diffusion and transport of nutrients^52^ and the subsequent cell colonization and proliferation. The complex hierarchy of bone allows for several levels of permeability provided by several porosity scales such as the trabecular spaces, the lacunae, or the canaliculi.^53,54^ Owing to the trabecular mimicking nature of the TPMS used in this study, the permeability values have been compared to those neglecting submicron bone structures. The permeability values obtained are in accordance to previously reported values for cancellous bone.^55^ Permeability varied from 0.002 mm^2^ for the lowest porosities to 0.02 mm^2^. The increase in the porosity yielded an increase in the permeability values (data not shown). Gyroid structures exhibited a highly linear dependency (*R*^2^ = 0.994), followed by Diamond and Schwarz (*R*^2^ = 0.927, and *R*^2^ = 0.901, respectively). Similar structures were investigated by Castro et al. finding slightly larger values of permeability ranging from 0.1 to 0.02 mm^2^, depending on the flow rate applied. Investigating 70% porous Schwarz and Gyroid produced using a photocurable polymer resin, they showed a dependency of permeability with the flow rate applied, which decreased inversely, and may explain the different values obtained in the present work.^17^ Zhang et al. also reported similar permeability values for graded porosities based on diamond geometries.^49^

TPMS have gained scientific attention thanks to their outstanding mechanical properties despite their porous structure. When aiming at regenerative scaffolds that eventually will be replaced by bone tissue, mechanical properties are often granted with help of external fixations. The elastic moduli and compressive strengths reported in this work exceeded the values of cancellous bone^56^ even for 65% porosities (Figure 6(b)) albeit still far from cortical bone values which have only been reported for metallic TPMS structures. Abueidda and Afshar et al. found similar mechanical properties for polymeric Gyroid and Schwarz structures with graded porosities, ranging from 80 to 500 MPa (global elastic moduli) and 5 to 27 MPa (compressive strength).^18,33^ Higher values, in the range of cortical bone, have been achieved using titanium alloys with 75% to 90% porous Gyroids.^35^ A similar study using PLA-based materials produced Gyroid and Diamond structures by SLA with 67% porosity, structures which exhibited elastic moduli from 170 to 324 MPa, that is, in the same order of magnitude as similar structures within this study.^15^

The TPMS structures investigated, although displaying the same total porosity, exhibited pore sizes ranging from 600 to 1400 µm, values ascribed to be necessary for bone regeneration in vivo.^7–9^ In vitro investigations have shown lower values of pore sizes to be beneficial, though differences have been pointed out depending on the surface chemistry. For instance, ceramic substrates have shown optimal pore size for bone formation from 75 to 100 µm,^57^ while metallic scaffolds yielded better performance for pores between 300 and 600 µm,^58,59^ similarly to polymeric materials.^60^ Interestingly, less reported parameters such as the interconnectivity and the pore size distribution have been linked to varying biological outcomes. Each of the TPMS studied exhibited a particular pore size distribution while maintaining similar interconnectivity levels (Figure 5(c), (d), (e), and (b), respectively). Diamond structures exhibited the highest interconnectivity levels among all geometries and monomodal pore size distributions. When correlating these parameters to the ALP levels observed in the biological studies, Diamond structures exhibited the lowest variations, with a quite steady and sustained expression of ALP (Figure 8). On the contrary, similar interconnectivity levels to bone,^24,30^ as those shown by Schwarz and Gyroid structures, exhibited a higher ALP expression after 7 days. Schwarz geometry, despite showing lower cell attachment at earlier time points, depicted a higher absolute increase in the ALP, especially for higher porosities, 50% and 65%. Furthermore, the correlation of pore size, interconnectivity, and differentiation (Figure 9) features a higher dependency on pore sizes than interconnectivity for cell differentiation. In fact, monomodal pore size distributions attributed to Diamond structures yielded the lowest ALP differentiation values compared to bimodal or multimodal from Gyroid and Schwarz, respectively. All geometries depicted low seeding efficiencies as porosity increased (Figure 7(b)). Nevertheless, cell proliferation was more pronounced for higher porosities (65%) after 7 days of cell culture. Murphy et al. demonstrated higher cell attachment for smaller pore sizes (120 µm) which reversed as culture time prolonged, showing higher cell viability for pore sizes larger than 300 µm.^60^ Likewise, Van Bael et al. pointed at higher seeding efficiencies for lower permeability scaffolds using selective laser melted titanium alloys,^61^ similarly to the present study for 35% porosities, and especially for Schwarz geometries. Furthermore, they also demonstrated that cell metabolic activity was enhanced for pore sizes of 1000 µm compared to 500 µm analogues, regardless of pore shape. Lower seeding efficiencies with open pores as those observed in the present study have been also reported by Ostrowska et al. using FDM printed polycaprolactone scaffolds.^62^ ALP was more constantly expressed on multimodal pore distributions as those of Schwarz structures, compared to bimodal ones from Gyroid, regardless of the porosity of the structures. In a recent in vivo study, Schwarz-based porous graded titanium alloy scaffolds produced by SLM demonstrated good osteointegration in tibial defects in mini pigs, highlighting the promising features of this particular TPMS structure.^63^ Overall, these results suggest that multimodal pore size distributions may have a higher interference in cell fate when interconnectivity is guaranteed.

## Conclusion

Overall, the results reported show that TPMS structures can be efficiently created with a low cost and highly available additive-manufacturing technology such as FDM. Despite the lower resolution of this technique, the morphometric analyses show good reproducibility and accuracy of the experimental and theoretical values. Furthermore, the mechanical and permeability properties of such constructs gave values within those of cancellous bone. Last but not least, preliminary biological data show that tuning the pore size and interconnectivity can boost the differentiation of pre-osteoblastic cell lines in spite of lower seeding efficiencies. Further studies improving the cell attachment efficiency may shed more light on the absolute effect of porosity traits on cell attachment.

## Supplemental Material

FigS1 – Supplemental material for Porous polylactic acid scaffolds for bone regeneration: A study of additively manufactured triply periodic minimal surfaces and their osteogenic potential Click here for additional data file. Supplemental material, FigS1 for Porous polylactic acid scaffolds for bone regeneration: A study of additively manufactured triply periodic minimal surfaces and their osteogenic potential by Anna Diez-Escudero, Hugo Harlin, Per Isaksson and Cecilia Persson in Journal of Tissue Engineering
