# Supplementary figures and images for: Porous polylactic acid scaffolds for bone regeneration: A study of additively manufactured triply periodic minimal surfaces and their osteogenic potential
Source: J Tissue Eng. 2020 Nov 6;11:2041731420956541. doi: 10.1177/2041731420956541 (PMC7656876; doi:10.1177/2041731420956541)

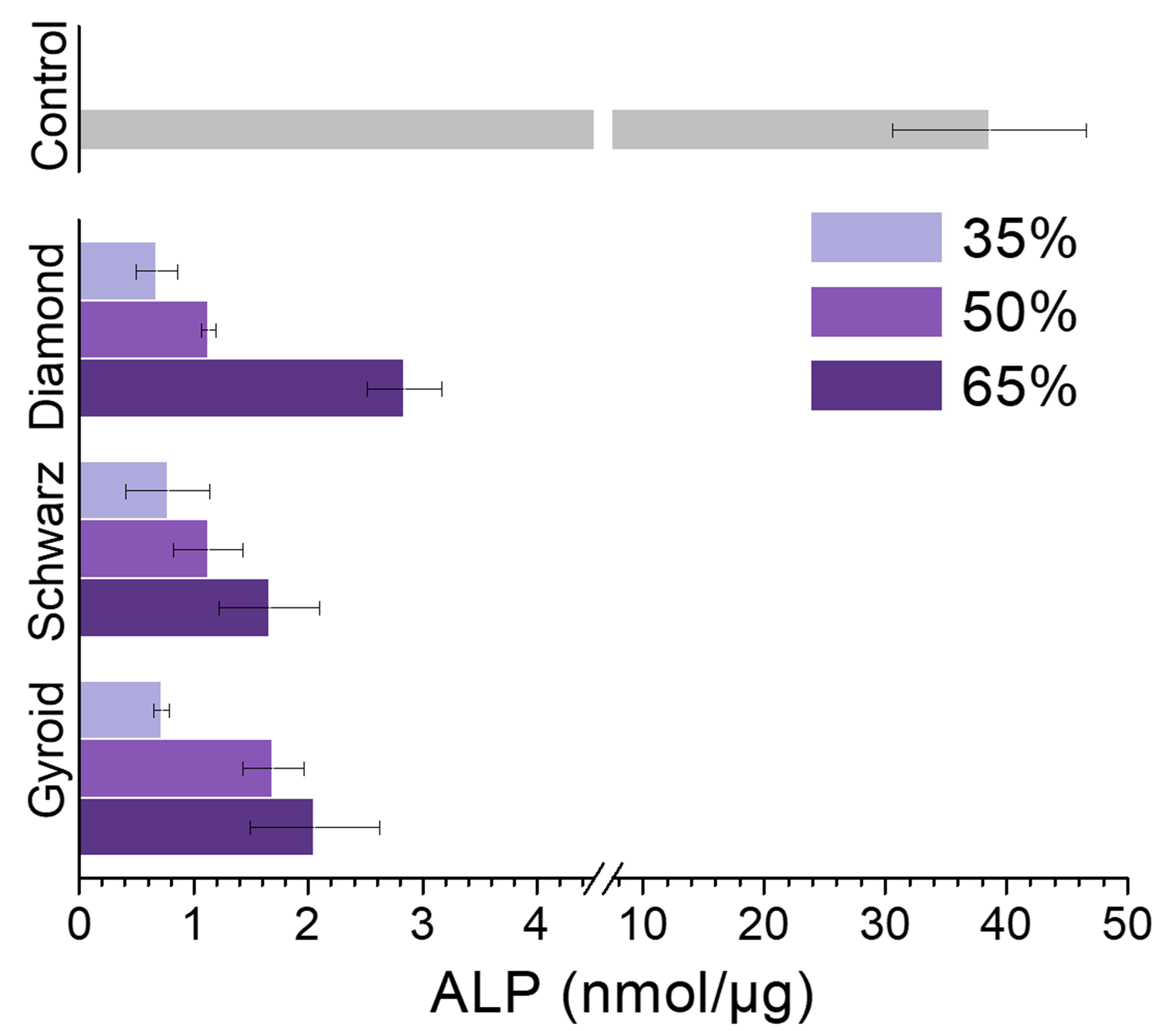

Supplement: FigS1 – Supplemental material for Porous polylactic acid scaffolds for bone regeneration: A study of additively manufactured triply periodic minimal surfaces and their osteogenic potential [file FigS1.tif]
